# Effectiveness of inactivated SARS-CoV-2 vaccines against the Delta variant infection in Guangzhou: a test-negative case–control real-world study

**DOI:** 10.1080/22221751.2021.1969291

**Published:** 2021-09-02

**Authors:** Xiao-Ning Li, Yong Huang, Wen Wang, Qin-Long Jing, Chun-Huan Zhang, Peng-Zhe Qin, Wei-Jie Guan, Lin Gan, Yi-Lan Li, Wen-Hui Liu, Hang Dong, Yu-Tian Miao, Shu-Jun Fan, Zhou-Bin Zhang, Ding-Mei Zhang, Nan-Shan Zhong

**Affiliations:** a Guangzhou Center for Disease Control and Prevention, Institute of Public Health, Guangzhou Medical University & Guangzhou Center for Disease Control and Prevention, Guangzhou, People’s Republic of China; b School of Public Health, Sun Yat-sen University, Guangzhou, People’s Republic of China; c National Centre for Respiratory Medicine, State Key Laboratory of Respiratory Disease & National Clinical Research Center for Respiratory Disease, Guangzhou Institute of Respiratory Health, The First Affiliated Hospital of Guangzhou Medical University, Guangzhou, People’s Republic of China; d Department of Thoracic Surgery, Guangzhou Institute of Respiratory Disease, First Affiliated Hospital of Guangzhou Medical University, Guangzhou, Guangdong, China

**Keywords:** Coronavirus disease 2019, SARS-CoV-2, efficacy of vaccination, Delta variant, vaccine

## Abstract

The effectiveness of inactivated SARS-CoV-2 vaccines against the Delta variant, which has been associated with greater transmissibility and virulence, remains unclear. We conducted a test-negative case–control study to explore the vaccine effectiveness (VE) in real-world settings. We recruited participants aged 18–59 years who consisted of SARS-CoV-2 test-positive cases (*n* = 74) and test-negative controls (*n* = 292) during the outbreak of the Delta variant in May 2021 in Guangzhou city, China. Vaccination status was compared to estimate The VE of SARS-CoV-2 inactivated vaccines. A single dose of inactivated SARS-CoV-2 vaccine yielded the VE of only 13.8%. After adjusting for age and sex, the overall VE for two-dose vaccination was 59.0% (95% confidence interval: 16.0% to 81.6%) against coronavirus disease 2019 (COVID-19) and 70.2% (95% confidence interval: 29.6–89.3%) against moderate COVID-19 and 100% against severe COVID-19 which might be overestimated due to the small sample size. The VE of two-dose vaccination against COVID-19 reached 72.5% among participants aged 40–59 years, and was higher in females than in males against COVID-19 and moderate diseases. While single dose vaccination was not sufficiently protective, the two-dose dosing scheme of the inactivated vaccines was effective against the Delta variant infection in real-world settings, with the estimated efficacy exceeding the World Health Organization minimal threshold of 50%.

## Introduction

The pandemic of coronavirus disease 2019 (COVID-19), caused by the severe acute respiratory syndrome coronavirus 2 (SARS-CoV-2) wild-type strain and its variants, has fuelled urgent needs to accelerate the development of vaccines to contain further spreading. Through concerted global efforts, several SARS-CoV-2 vaccines (i.e. inactivated vaccine, recombinant protein vaccine, adenovirus vector vaccine, DNA vaccine and RNA vaccine) have been developed. These vaccines have achieved the vaccine efficacy (VE) of up to 95% in clinical trials [1–3]. However, concerns have been raised regarding the reduced VE against the emerging variants globally [4]. Compared with the wild-type strain, the D614G mutation of the spike protein was more transmissible, and the B.1.1.7 strain elicited a higher rate of hospitalization and mortality in the UK [5]. The VE of ChAdOx1 nCoV-19 vaccine against the B.1.351 variant (first emerged in South Africa) declined from 89.3% to 21.9% in a phase 3 trial, and to 62% in the combined analysis of the randomized controlled trials [6–8]. Interim results from a phase III trial indicated the VE of Novavax vaccine against the B.1.1.7 and B.1.351 variants of 85.6% and 60%, respectively, although the overall VE reached to 95.6% against the wild-type strain [9]. In another trial of NVX-CoV2373 vaccine against the B.1.351 variant, the VE was only 49.4% [10]. These findings have confirmed the decreased efficacy of various vaccines against the emerging variants.

Currently, the COVID-19 vaccination has been enforced in more than 200 countries and regions to contain further SARS-CoV-2 transmissions. Two inactivated vaccines, the China National Biotec Group SARS-CoV-2 vaccine and the CoronaVac vaccine (Sinovac Biotech Ltd., China) have been adopted for mass vaccination within mainland China. Animal experiments and phase 1and 2 clinical trials have consistently demonstrated a low rate of adverse reactions and notable immunogenicity with potent protection against the virus challenge in non-human primates [11–14]. However, phase 3 clinical trials have not been available due to the effective control of the local epidemic. In a real-world study in Chile spanning from February 2 through May 1, 2021, the effectiveness of CoronaVac was estimated to be 65.9% for the prevention of COVID-19 and 87.5% for the prevention of hospitalization, 90.3% for the prevention of intensive care unit (ICU) admission, and 86.3% for the prevention of COVID-19–related deaths [15].

To improve the preparedness against future epidemics, 1049 million doses of inactivated SARS-CoV-2 vaccines have been vaccinated in mainland China as of June 21, 2021. Since mid-May 2021, an emerging COVID-19 outbreak associated with the Delta variant (the B.1.617.2 variant) has emerged in Guangzhou city. Understanding the efficacy of SARS-CoV-2 vaccines against the Delta strain has become the top priority. Results from the Public Health England indicated that the VE of ChAdOx1 nCoV-19 vaccine and BNT162b2 vaccine against the Delta strain was 71% and 94% after a single dose and 92% and 96% after two doses [16], respectively. Another study found that the neutralization of the Delta strain was reduced compared with the wild-type strain *in vitro* [17].

During the recent outbreak in Guangzhou, all cases have been linked to the first patient possibly transmitted by an imported case. All close contacts were further isolated and monitored for 14–21 days. Meanwhile, all residents were subject to SARS-CoV-2 nucleic acid detection at regular intervals to fully identify the potential sources of infection. Some infected residents with COVID-19 and their contacts have been vaccinated. Therefore, these scenarios have provided a rare opportunity to evaluate the VE of SARS-CoV-2 inactivated vaccine against the Delta variant in the real-world setting in Guangzhou.

## Methods

### Study design and population

In this test-negative case–control study, we estimated the VE of two SARS-CoV-2 inactivated vaccines against the infection or pneumonia associated with the Delta strain. All study participants were residing in Guangzhou, the provincial capital city with a population of 18,676,600. We have only included the population aged 18–59 years given their priority to be vaccinated according to the national policy as of June 2021. Study participants were stratified into two groups: SARS-CoV-2 test-positive cases (cases) and test-negative controls. Cases and controls were selected among the COVID-19 patients and close contacts of cases who had a high probability of contracting the virus. Ethics approval was waived given the need to collect data from routine observations.

### Case and close contacts definition

Upon confirmation of the case (regardless of the presence of symptoms), an immediate submission of the medical records to the Guangzhou Center for Disease Control and Prevention (GZCDC) was mandatory. After the receipt of a submitted report, the GZCDC performed an epidemiological investigation within 12 h, including verification of the activity trajectory, close contact determination and tracing.

SARS-CoV-2 test-positive cases were identified through screening of suspected symptomatic cases in medical institutions, residents of the community where the previous case was located, or close contacts of the previous case. The cases consisted of the patients with COVID-19 diagnosed between May 18 and June 20, 2021 at medical institutions in Guangzhou and followed the same diagnostic criteria.

Close contacts denoted the residents who were in contact (without an effective protection, such as jointly living, eating and working in the same room, traveling, seeking clinician’s consultations, taking the elevator or attending social activities without wearing mask) with the SARS-CoV-2 test-positive cases in the same confined space at any time, starting from 4 days before the onset of symptoms of the confirmed patients, or from 4 days before sampling of asymptomatic patients. Close contacts were centrally quarantined for 14 days after the last unprotected close contact with the confirmed cases and asymptomatic patients, followed by home quarantine for 7 days. During the quarantine period, nasopharyngeal swabs of close contacts were taken by trained medical staffs for RT-PCR assay of SARS-CoV-2 nucleic acid daily of the first week and at days 10, 14, 16 and 21. Close contacts were released from quarantine if the real-time polymerase chain reaction (RT-PCR) assay of SARS-CoV-2 nucleic was consistently negative during this period.

In this outbreak, we estimated the number of close contacts would exceed 10,000. To ensure the comparability between cases and controls and avoid selection bias, we selected all the 18–59-year-old close contacts with a higher frequency of contact (jointly living, eating, or working) as the controls. Both cases and controls were likely to have the same extent of experience in their exposure to SARS-CoV-2.

### Case classification

Confirmed cases were classified as having mild, moderate, severe and critical illness according to the latest edition of the national diagnosis and treatment protocol for COVID-19 in China. Mild COVID-19 denoted the cases without signs of pneumonia on chest imaging. Cases with fever, respiratory symptoms and imaging characteristics of pneumonia were classified as having moderate COVID-19. Severe COVID-19 met any of the criteria: respiratory rate >30/min, resting oxygen saturation <93%, and oxygenation index <300 mmHg. Critical COVID-19 cases were those with respiratory failure and requiring mechanical ventilation, shock or other organ failure requiring admission to the ICU.

### Information collection

The demographic (age, gender, occupation), SARS-CoV-2 vaccination (date of vaccination and manufacturer), epidemiology (exposure date, relationship with other cases and contacts, contact frequency, contact place, contact mode, sampling date and results) and clinical characteristics (symptoms, severity classification, date of onset) were collected by the study investigators.

Because two weeks were required to form the protective effects against SARS-CoV-2 infections, we defined the first-dose vaccination (partially vaccinated) and second-dose vaccination (fully vaccinated) as having elapsed for more than 14 days after the first dose or second dose upon the clinical diagnosis (for cases) or the last contact with the cases (for contacts). Otherwise, study participants would be deemed non-vaccinated despite that they had received the first dose of vaccination, and deemed having received the first dose of vaccination only although they had received the second dose.

### Statistical analysis

The sample size was estimated based on the formula adapted to the case–control study, $n=2\bar{\;pq}(Z_{\alpha }+Z_{\text{β}}{)}^{2}/(\;p_{1}-p_{0}{)}^{2}$. The threshold of *α* was set at 0.05 and *β* at 0.10. The rate of protection (*p*_1_) by the vaccine was estimated to be 70%, and the vaccination rate of the control group (*p*_0_) was estimated to be 60%. Therefore, the minimum sample size was 124 (62 per group) to provide a statistical power of 90%.

All data were analyzed with the *R* Statistical Software 3.6.3. Categorical and continuous variables were compared by using the Chi-squared test and *t*-test, respectively. We initially compared the characteristics of the vaccinated and unvaccinated cases. The VE corresponding to partial vaccination was estimated to be VE_1 dose_ = (1−OR_1 dose_) × 100% and the two-dose VE was estimated to be VE_2 dose_ = (1−OR_2 dose_) × 100%, where the odds ratio (OR) was derived from a logistic regression model, which had been adjusted for the age and gender. To determine whether the VE could also be affected by the age and sex, we performed subgroup analysis by stratification according to the age (cut-off: 40 years) and sex. The level of statistical significance was defined as *P* < 0.05.

## Results

### Study participant recruitment

**Figure 1.**
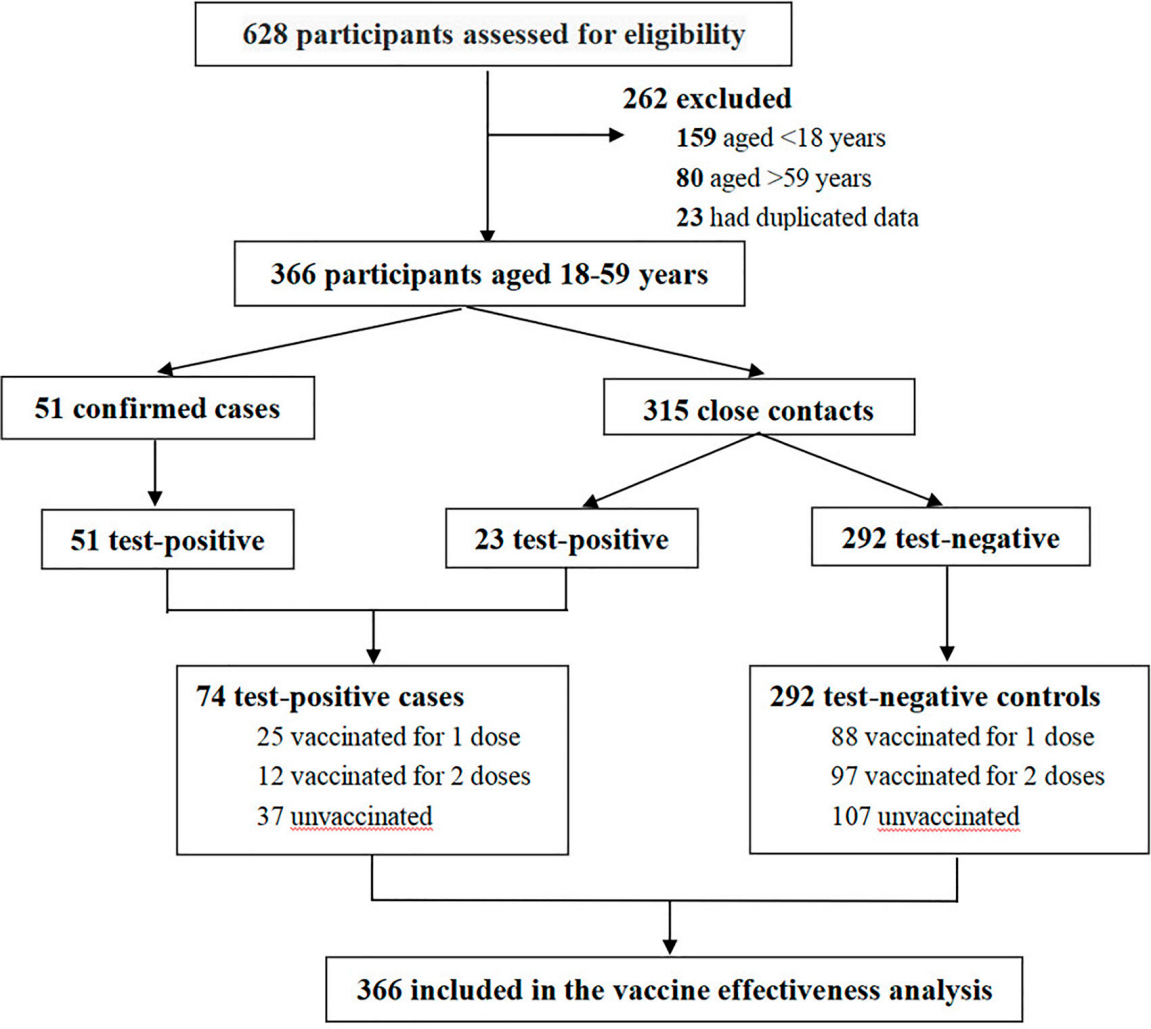
Flowchart of selection of study participants.

A total of 153 cases were confirmed inside Guangzhou city, and therefore, the information of 628 study participants (along with 475 close contacts) was collected. Two hundred and sixty-two study participants were excluded because they were outside the age range (18–59 years) for vaccination (*N* = 239), or were duplicate participants (*N* = 23). Seventy-four test-positive cases and 292 test-negative controls were included in the final analysis. The trial profile is shown in Figure 1.

### Characteristics of cases and controls

Of the 153 confirmed cases, 32 had mild COVID-19, 105 had moderate COVID-19, 6 had severe COVID-19, and 10 had critical COVID-19. There were no deaths during the study period. None of the 16 cases with severe and critical COVID-19 have been vaccinated with SARS-CoV-2 vaccines.

The demographic and clinical characteristics of vaccinated and unvaccinated cases were shown in Table 1. Most vaccinated cases (97.4%) were aged 18–59 years. 59.5% of cases (*N* = 91) reported symptoms within 3 days after viral RNA detection. Symptoms appeared at a median of 29 days (IQR: 17.8∼57.0) after vaccination. 57.9% of patients had an interval of greater than 28 days between vaccination and onset of symptoms. No significant difference in this interval was observed when stratified by the age, sex, clinical severity, the cycle threshold (Ct) of RT-PCR assays, symptoms and time from onset to clinical cure. One hundred and twenty-three of the 153 cases were subject to the second- and third-generation sequencing of respiratory specimens, with the final results indicating that all isolated strains were the Delta variants.

**Table 1.** Characteristics of all the cases identified in the outbreak in Guangzhou.

	Overall (*n* = 153)	Vaccinated (*n* = 38)	Unvaccinated (*n* = 115)	*p*-Value
*Age*				
Median [IQR]	48.0 [30.0, 67.0]	45.5 [39.5, 51.7]	56.0 [21.5, 71.5]	0.157
Age group (years)				<0.001
≤17	26 (17.0%)	0	26 (22.6%)	
18∼59	74 (48.4%)	37 (97.4%)	37 (32.2%)	
≥60	53 (34.6%)	1 (2.6%)	52 (45.2%)	
*Gender*				0.806
Male	63 (41.2%)	15 (39.5%)	48 (41.7%)	
Female	90 (58.8%)	23 (60.5%)	67 (58.3%)	
*Clinical severity*				0.009
Mild	32 (21.0%)	6 (15.8%)	26 (22.6%)	
Moderate	105 (68.6%)	32 (84.2%)	73 (63.5%)	
Severe	6 (3.9%)	0	6 (5.2%)	
Critical	10 (6.5%)	0	10 (8.7%)	
*Time from symptom onset to viral RNA detection, days*				0.957
−3 to 0	91 (59.5%)	23 (60.5%)	68 (59.1%)	
1–3	47 (30.7%)	11 (29.0%)	36 (31.3%)	
≥4	15 (9.8%)	4 (10.5%)	11 (9.6%)	
*Time from vaccination to symptom onset, days (Median, IQR)*	29 (17.8∼57.0)	29 (17.8∼57.0)	–	
*Time from vaccination to hospitalisation, days*				–
≤14	6 (15.8%)	6 (15.8%)	–	
15–27	10 (26.3%)	10 (26.3%)	–	
≥28	22 (57.9%)	22 (57.9%)	–	
*Time from onset to clinical cure, days*				0.079
≤10	4 (2.6%)	0	4 (3.48%)	
11–19	48 (31.4%)	17 (44.7%)	31 (27.0%)	
≥20	101 (66.0%)	21 (55.3%)	80 (69.7%)	
*PCR cycle threshold (ct value)*				0.226
<24	74 (48.4%)	17 (44.7%)	57 (49.6%)	
24–40	62 (40.5%)	20 (52.6%)	42 (36.5%)	
*Symptoms*				
Fever	61 (39.87%)	20 (52.6%)	41 (47.4%)	0.064
Cough	41 (26.8%)	12 (31.6%)	29 (25.2%)	0.443

Note: *p*-Values were estimated by *χ*² tests or *t*-test. The “–” indicated no data.

Time from vaccination to hospitalisation: Some of the patients in our study were hospitalized after the onset of illness, and the remaining were hospitalized after large-scale nucleic acid screening (who might not have developed clinical symptoms at screening) for once the cases were confirmed as SARS-CoV-2 positive, admission to the designated hospital was mandatory.

The clinical characteristics of 74 cases and 292 controls are demonstrated in Table 2. Cases were markedly older than controls (median: 44 vs. 39 years, *P* = 0.005). Participants aged 18–29 years accounted for 30.1% of controls. 177 (60.6%) were males in the control group while 29 (39.2%) were males among the cases. Individuals with moderate disease accounted for 81.1% of the cases.

**Table 2.** Characteristics of SARS-CoV-2 cases and controls for vaccine effectiveness estimation.

	Test-positive cases (*n* = 74)	Test-negative controls (*n* = 292)	*p* Value
*Age*			
Median [IQR]	44.0 [36.2, 51.7]	39.0 [27.0, 48.0]	0.005
Age group (years)			0.055
18–29	12 (16.3%)	88 (30.1%)	
30–45	32 (43.2%)	108 (37.0%)	
46–59	30 (40.5%)	96 (32.9%)	
*Gender*			0.001
Male	29 (39.2%)	177 (60.6%)	
Female	45 (60.8%)	115 (39.4%)	
*Clinical severity*			
Mild	12 (16.2%)	–	
Common	60 (81.1%)	–	
Severe	2 (2.7%)	–	
Critical	0	–	
*SARS-CoV-2 vaccination history*			0.024
Unvaccinated	37 (50.0%)	107 (36.6%)	
1 dose < 14 days	7 (9.5%)	40 (13.7%)	
1 dose ≥ 14 days	18 (24.3%)	48 (16.4%)	
2 doses < 14 days	2 (2.7%)	18 (6.2%)	
2 doses ≥ 14 days	10 (13.5%)	79 (27.1%)	

Note: *p*-Values were estimated by *χ*² tests or *t*-test. The “–” indicated no data.

The vaccination status was disproportionate between cases and controls who received vaccination of two doses (16.2% vs. 33.3%; *P* = 0.024) but not a single dose (33.8% vs. 30.1%; *P* > 0.05). Of these, 18 (24.3%) cases and 48 (16.4%) controls were vaccinated with the first dose for more than 14 days, and 10 (13.5%) cases and 79 (27.1%) controls were vaccinated with the second dose for more than 14 days. The vaccines consisted of both the CoronaVac vaccine or China National Biotec Group (CNBG) SARS-CoV-2 inactivated vaccine, and most study participants were vaccinated with CoronaVac vaccine (136, 61.3%). There were 61 (27.5%) study participants who were vaccinated with CNBG vaccine, 23 (10.4%) study participants were vaccinated with both vaccines, and 2 (0.8%) participants had missing information of the vaccination type. Therefore, 175 people more than 14 days after vaccination were finally included in the analysis of VE.

### SARS-CoV-2 vaccine effectiveness

**Figure 2.**
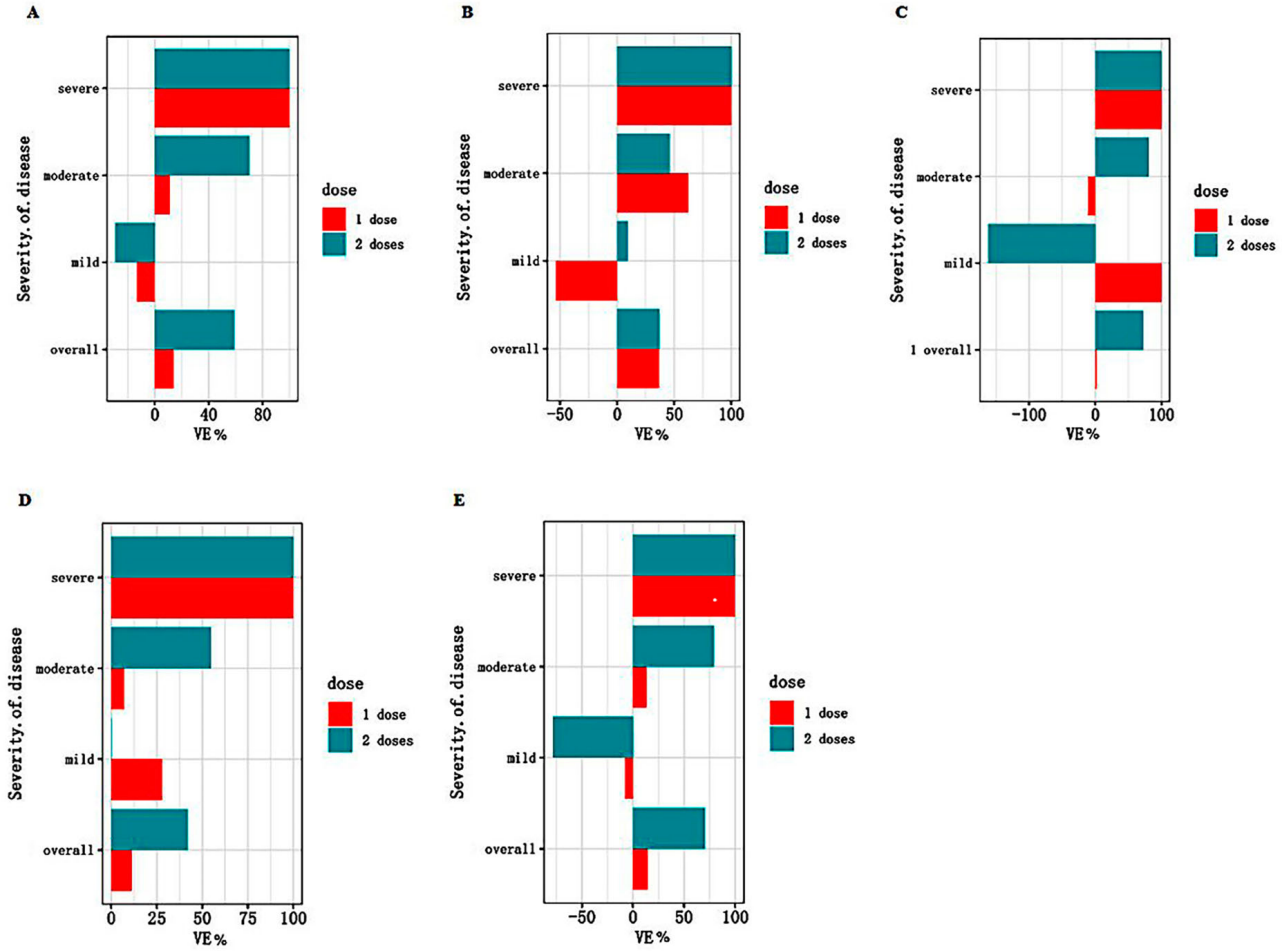
Effectiveness of the inactivated vaccines against different severity of COVID-19 associated with the Delta variant strain. (A) Effectiveness in the whole population. The abscissa is VE (%) and the ordinate are different severity of clinical manifestations. The bars represent the estimated value of VE. Different colors indicate different vaccination doses. (B) Effectiveness in the study participants aged 18–39 years. (C) Effectiveness in study participants aged 40–59 years. (D) Effectiveness in males. (E) Effectiveness in females.

In unadjusted analysis, the VE for a single dose vaccine was −1% (95%CI: −83% to 55%) and the VE for two doses of vaccination was estimated to be 58% (95%CI: 15% to 81%). After adjusting for the age and sex, a single dose of vaccine (VE: 13.8%, 95%CI: −60.2% to 54.8%) was not sufficiently protective against COVID-19, as did mild, moderate and severe COVID-19 (critical COVID-19 was not included because there were no critical cases among the 74 cases we have analyzed). The VE for the two-dose full vaccination was estimated to be 59.0% (95%CI: 16.0% to 81.6%) against COVID-19, 70.2% (95%CI: 29.6% to 89.3%) against moderate COVID-19. The VE against the severe disease was estimated as 100% because there were two severe cases who were unvaccinated in the case group, which might be overestimated due to the small samples. The VE of different doses had been shown in Figure 2 and the VE for the two-dose full vaccination was markedly higher than that for the single-dose (partial) vaccination.

We have further analyzed the VE by stratification of the study participants with the age. The protective effect of the two-dose full vaccination against the Delta strain reached 72.5% (95%CI: 23.9% to 91.6%) among the study participants aged 40–59 years. Next, we analyzed the VE when stratified by sex. Compared with the overall analyses, the VE of two doses against COVID-19 (70.4%, 95%CI: 18.4% to 91.0%) and moderate COVID-19 (79.1%, 95%CI: 30.9% to 95.4%) was consistently higher in females than in males (Table 3).

**Table 3.** Estimates of the association between vaccination and SARS-CoV-2 infection and effectiveness of inactivated SARS-CoV-2 vaccines.

	Vaccinated	Unvaccinated	1 dose OR_adj_ (95%CI)	1 dose VE_adj_ (%, 95%CI)	Vaccinated	Unvaccinated	2 doses OR_adj_ (95%CI)	2 doses VE_adj_ (%, 95%CI)
*The whole population*								
Control	66	147	Reference	–	79	147	Reference	–
Overall cases	20	44	0.86 (0.45–1.60)	13.8 (−60.2, 54.8)	10	44	0.41 (0.19–0.84)	59.0 (16.0, 81.6)
Mild cases	2	6	1.13 (0.16–5.53)	−13.4 (−452.5, 84.3)	4	6	1.29 (0.32–4.69)	−29.4 (−369.4, 67.9)
Moderate cases	18	36	0.89 (0.45–1.73)	11.2 (−72.5, 55.5)	6	36	0.30 (0.11–0.70)	70.2 (29.6, 89.3)
Severe cases	0	2	–	–	0	2	–	100
*18–39 years*								
Control	28	86	Reference	–	39	86	Reference	–
Overall cases	4	18	0.63 (0.17–1.90)	36.9 (−89.9, 83.1)	5	18	0.63 (0.20–1.74)	37.0 (−73.9, 80.5)
Mild cases	2	5	1.54 (0.21–8.14)	−53.7 (−714.3, 79.5)	2	5	0.91 (0.13–4.47)	9.3 (−346.9, 87.5)
Moderate cases	2	13	0.38 (0.06–1.54)	62.2 (−54.1, 94.5)	3	13	0.54 (0.12–1.85)	46.1 (−85.2, 88.4)
Severe cases	0	0	–	–	0	0	–	–
*40–59 years*								
Control	38	61	Reference	–	40	61	Reference	–
Overall cases	16	26	0.98 (0.44–2.15)	1.8 (−115.3, 56.0)	5	26	0.26 (0.08–0.76)	72.5 (23.9–91.6)
Mild cases	0	1	–	–	2	1	2.62 (0.23–59.03)	−161.9 (−5803.3, 76.8)
Moderate cases	16	23	1.11 (0.49–2.47)	−11.0 (−147.3, 50.8)	3	23	0.19 (0.04–0.63)	80.6 (37.1, 95.7)
Severe cases	0	2	–	–	0	2	–	100
*Males*^a^								
Control	40	91	Reference	–	46	91	Reference	–
Overall cases	7	17	0.89 (0.32–2.27)	11.2 (−126.7, 68.2)	5	17	0.58 (0.18–1.58)	41.9 (−57.5, 81.8)
Mild cases	1	4	0.72 (0.04–5.39)	27.8 (−438.9, 96.4)	2	4	1.00 (0.13–5.37)	−0.1 (−437.4, 86.6)
Moderate cases	6	13	0.93 (0.30–2.58)	7.2 (−157.6, 69.8)	3	13	0.45 (0.10–1.49)	54.8 (−49.2, 90.0)
Severe cases	0	0	–	–	0	0	–	–
*Females*^a^								
Control	26	56	Reference	–	33	56	Reference	–
Overall cases	13	27	0.85 (0.36–1.98)	14.6 (−97.8, 64.3)	5	27	0.30 (0.09–0.82)	70.4 (18.4, 91.0)
Mild cases	1	2	1.08 (0.05–11.74)	−8.0 (−1074.0, 95.0)	2	2	1.78 (0.20–15.76)	−78.4 (−1476.2, 79.7)
Moderate cases	12	23	0.87 (0.35–2.11)	13.2 (−111.2, 65.5)	3	23	0.21 (0.05–0.69)	79.1 (30.9, 95.4)
Severe cases	0	2	–	–	0	2	–	100

Note: OR were adjusted for age and gender. The “–” indicated no data.

^a^ OR only adjusted for age.

## Discussion

The high transmissibility and markedly increased virulence of the Delta variant strain have resulted in a considerable socioeconomic burden, causing an enormous stress on the community-level containment. Prioritization has been given to the urgent development and validation of vaccines against the emerging virulent strain. Because of the effective containment of the epidemic before the current outbreak, testing of the VE has been challenging within mainland China. Therefore, the scenario of the real-world setting in this study has offered an opportunity to determine the VE of two prevailing inactivated vaccines in mainland China against the emerging Delta strain.

Our study has demonstrated the estimated VE of two doses of inactivated SARS-CoV-2 vaccine of 59.0% against the Delta variant infection. Overall, the VE was lower compared with that previously reported (∼79.3%) [18]. By contrast, in our study, the VE against moderate COVID-19 was higher than mild COVID-19. The VE against the severe disease was estimated as 100%. Since there were only 2 severe cases in the case group, the VE against severe cases might be overestimated in this study. However, in this outbreak, there were no severe and critical cases or deaths among the vaccinated study participants, and all the 16 severe or critical cases were not vaccinated. Therefore, we speculated that the inactivated SARS-CoV-2 vaccine could prevent severe COVID-19 well. The World Health Organization has set a minimal threshold of 50% for the VE of all SARS-CoV-2 vaccines [19]. Therefore, our findings indicated that full vaccination with two doses of the inactivated SARS-CoV-2 vaccines was effective against the Delta variant.

The epidemic prevention measures against COVID-19 adopted in China had been successful. However, in light of the need to prevent from imported cases, mass vaccination was progressively launched. To safeguard the supply of vaccines to the community (especially the major workforce) and vaccination safety, priority was given to the population aged 18–59 years. The findings that the VE of two-dose vaccination against the Delta strain was greater than 72.5% among the study participants aged 40–59 years, and that the VE of two-dose vaccination was higher in females than in males, indicating the need to further strengthen vaccination of the elderly and females during the ongoing vaccination programme.

A single dose vaccine was not sufficiently protective against the Delta strain infection, which was consistent with the findings pertaining to the wild-type strains [20]. This was not surprising, particularly when viewing from the greater transmissibility and virulence of the Delta strain [21]. Moreover, because one dose of vaccination yielded a lower VE than two-dose vaccination, full vaccination should be recommended for community-based mass vaccination programmes.

We have investigated the two inactivated vaccines because they have been officially approved by the World Health Organization for emergency use. Our findings might have important global implications because more than 350 million doses of vaccines have been shared to the international community as of June 2021 [22]. Several studies have estimated the VE of inactivated vaccines in different countries. A study in Brazil [23] reported the VE of a single dose of CoronaVac vaccine to be 50.7% (95% CI: 35.6% to 62.2%) against the P.1 variant (B.1.1.7 strain), and another study in Chile showed that two doses of CoronaVac vaccine yielded an efficacy of 67% in preventing from symptomatic COVID-19 [15]. In our study, the effectiveness for two-dose vaccination was estimated to be 59.0% against COVID-19 and 70.2% against moderate COVID-19, indicating that the non-inferior protective effect of the inactivated vaccine on Delta virus compared with other variants and the wild-type strain.

We have determined the VE by using a test-negative case–control design, which has been widely used in vaccine evaluation [24–26]. However, the selection of controls might have affected the estimated VE, and the controls in our study had a higher frequency of contact with the cases compared with the general population. Our effect estimates of the VE should be better appreciated under the scenario of various concurrent stringent containment efforts enforced by the local government, which have collectively contributed to the effective control of the outbreak within only one month. Meanwhile, the vaccination status of the two inactivated vaccines was not balanced, and most participants were vaccinated with the CoronaVac vaccine, the predominant type of vaccine supplied by the local communities. However, the primary objective of our study was not to compare the VE between the two vaccines. Both vaccines consistently belonged to the inactivated vaccines and had been widely used throughout mainland China, and therefore data of these two vaccines have been combined for final analysis in our study. Finally, while observational studies might not be an ideal platform for estimating the VE as compared with large-scale randomized controlled trials, and the data provided in this study was only from a limited number of patients in one city and cannot be generalized. There remain no existing real-world study which delineated the protective effect of inactivated vaccines against the Delta strain. Although no firm conclusions could be extrapolated in other regions of the world, our findings have provided a valuable evidence indicating the VE against the Delta variant in real-world settings, and we hope the global scientific world will collaborate to disclose more similar data, so as to know the efficacy of different vaccines versus delta variants.

## Conclusion

A single dose of inactivated vaccines was not sufficiently protective against the Delta strain infection among the population aged 18–59 years. A two-dose dosing scheme yielded an overall VE of 59.0% against the Delta variant, with higher VE being noted in females and people aged 40-59. Although caution should be exercised when determining the generalizability, our findings have justified the need to continuously enforce mass vaccination against the Delta strain.
